# Targeting ferroptosis in spinal cord injury through stem cell therapy: mechanisms and therapeutic prospects

**DOI:** 10.3389/fnins.2025.1622787

**Published:** 2025-06-25

**Authors:** Qiqin Shen, Lingdi Wu, Chuyi Cai, Bingbing Li, Fang Yu

**Affiliations:** ^1^Department of Spine Surgery, Shaoxing Central Hospital, Affiliated Central Hospital of Shaoxing University, Shaoxing, Zhejiang Province, China; ^2^Department of Radiology, Ningbo No. 2 Hospital, Ningbo, Zhejiang Province, China; ^3^Department of Pediatrics, Shaoxing Central Hospital, Affiliated Central Hospital of Shaoxing University, Shaoxing, Zhejiang Province, China

**Keywords:** spinal cord injury, ferroptosis, stem cell therapy, programmed cell death, neural repair

## Abstract

Spinal cord injury (SCI) is a traumatic neurological disorder with a high incidence and limited clinical treatment options. Ferroptosis, a newly discovered form of programmed cell death, has shown significant research potential in the field of neurological diseases. Stem cells have become an ideal therapeutic option for various diseases due to their multidirectional differentiation potential and paracrine properties. Existing studies have demonstrated that stem cells possess substantial potential in the repair of spinal cord injuries. Recent research has found that stem cell transplantation can improve the pathological process of SCI by regulating the ferroptosis pathway. This review systematically described the molecular mechanisms of ferroptosis in SCI, the biological effects of stem cell therapy for SCI, and the therapeutic potential of stem cell-targeted regulation of ferroptosis. Additionally, we proposed three key research directions: cross-study of ferroptosis signaling pathways and stem cell action mechanisms, optimization strategies for therapeutic stem cells, and multimodal combined treatments based on ferroptosis regulation. This review aimed to provide new theoretical foundations and research perspectives for stem cell therapy in SCI.

## 1 Introduction

SCI is a serious disabling disease of the central nervous system, leading to motor, sensory, and autonomic dysfunction below the injury site (Anjum et al., 2020). Based on the pathological mechanism, SCI can be divided into primary and secondary injury: the former is caused by direct mechanical force and is usually irreversible, while the latter involves a cascade of dynamic regulatory reactions and plays a key role in the deterioration of neurological function (He et al., 2024). New research suggests that the primary reason for poor regeneration and functional recovery in SCI is “microenvironmental imbalance,” which manifests as an increase in inhibitory factors at the tissue, cell, and molecular levels, and a decrease in promoting factors at different times and locations (Fan et al., 2018). As the core pathological event of secondary injury, programmed cell death significantly impairs neurological function recovery and clinical prognosis by exacerbating microenvironmental damage and neuronal cell death, suggesting that its regulation may become a potential target for SCI treatment (Shi et al., 2021; He et al., 2024).

Epidemiological data show that the global prevalence of SCI has risen steadily over the past 30 years, with incidence rates in various countries ranging from 236 to 1,298 cases per million people (Khorasanizadeh et al., 2019). SCI was once considered an untreatable disease when it was first mentioned in the Edwin Smith Papyrus manuscript in the 17th century BC (Hughes, 1988). Although current clinical treatments primarily involve surgical intervention, drug therapy, hyperbaric oxygen, and physical therapy, the overall efficacy and prognosis remain unsatisfactory (Eli et al., 2021; Fehlings et al., 2017; Gao et al., 2020). This is mainly due to the lack of effective tools for regenerating neural tissue (Jendelova, 2018). Recent studies have identified ferroptosis, an iron-dependent form of programmed cell death driven by membrane lipid peroxidation (Jiang et al., 2021), as playing a crucial role in the pathological process of SCI (Shi et al., 2021). Ferroptosis is regulated by multiple pathways, including cysteine transport, glutathione metabolism, glutathione peroxidase 4 (*GPX4*) function, and *FSP1* protein, and its activation can lead to neuronal dysfunction and increased damage (Li et al., 2022; Ge et al., 2021). Traditional treatments are still ineffective in preventing the occurrence of ferroptosis (Song et al., 2023).

Given the challenges in SCI repair, stem cell therapy has shown unique advantages. Stem cells have significant potential in inhibiting ferroptosis and promoting neural regeneration by differentiating into neurons to replace damaged cells, secreting neurotrophic factors, and regulating the inflammatory microenvironment (Song et al., 2023; Shao et al., 2019). This strategy offers a new research direction for overcoming the existing treatment bottleneck. This review systematically discusses the role of stem cell therapy in SCI treatment, with a focus on ferroptosis as a therapeutic target.

## 2 Overview of stem cell therapy

**Figure 1:**
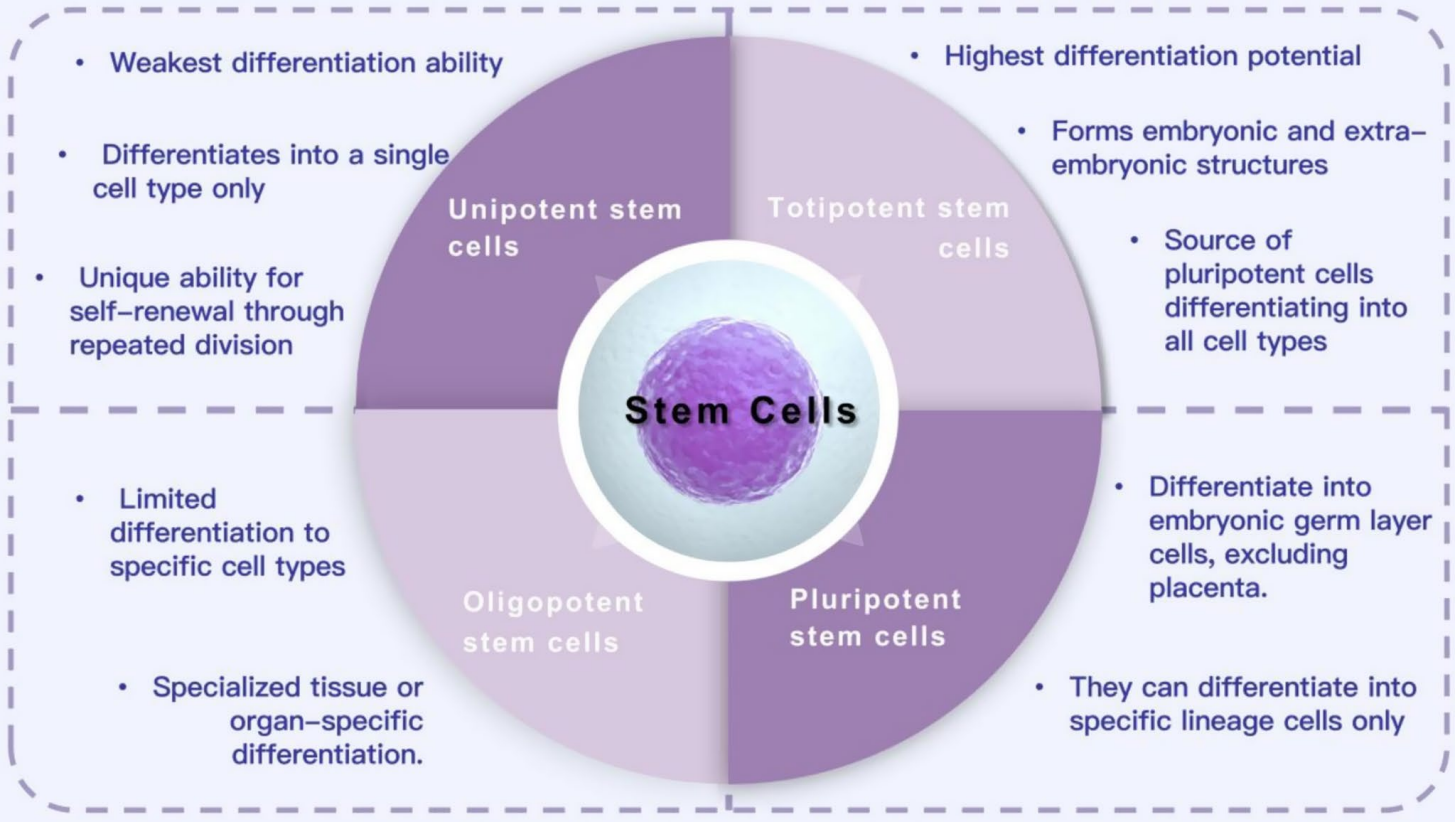
Classification and function of stem cells.

Stem cells are a group of cells with differentiation potential. They have the ability to self-renew, proliferate, and differentiate into various functional cell types through division. They are widely used to generate different tissues and organ systems (Tian et al., 2023). Stem cells come from various sources and have different differentiation potentials. Based on their differentiation ability, they are classified into totipotent, pluripotent, oligopotent, and unipotent stem cells (Kolios and Moodley, 2013; Zakrzewski et al., 2019). Totipotent stem cells have the highest differentiation potential and can form embryos and extraembryonic structures. Pluripotent stem cells (PSCs) can proliferate indefinitely and differentiate into cells of all three germ layers. These characteristics make PSCs an ideal option for cell therapy in various diseases and injuries (Yamanaka, 2020). Oligopotent stem cells can differentiate into multiple cell types, such as myeloid stem cells that can differentiate into white blood cells. Unipotent stem cells have the weakest differentiation ability and can only form one cell type, but they can divide repeatedly. Based on their sources, stem cells can be classified into various types, such as embryonic stem cells, umbilical cord stem cells, bone marrow mesenchymal stem cells, tendon stem cells, dental pulp stem cells, and adipose mesenchymal stem cells (Ankrum et al., 2014; Nombela-Arrieta et al., 2011; Hu et al., 2020; He et al., 2024; Zhang et al., 2023). Currently, stem cells are widely used in regenerative medicine for treating SCI, osteoarthritis, heart disease, retinopathy, diabetes, and other diseases, as well as in tissue engineering and drug testing (Li et al., 2019; Zhang et al., 2015; Mikłosz and Chabowski, 2023; Yu et al., 2022; Cofano et al., 2019; Figure 1).

Various stem cell populations have shown important application potential in SCI regenerative therapy due to their self-renewal characteristics and multi-lineage differentiation potential. Current preclinical research mainly focuses on three types of stem cells: mesenchymal stem cells (MSCs), neural stem cells (NSCs), and PSC-derived cells (Martin-Lopez et al., 2021). Among them, MSCs not only improve the pro-inflammatory microenvironment of the injury site through immunomodulatory effects (Cofano et al., 2019) but also repair neuronal mitochondrial function by mediating mitochondrial transfer, thereby inhibiting the progression of secondary injury (Yao et al., 2023). Additionally, exosomes secreted by MSCs, as intercellular communication mediators, can cross the blood-spinal cord barrier and achieve precise molecular regulation by delivering specific miRNAs, making them unique drug carriers (Liu et al., 2021). NSCs work through a dual mechanism: on one hand, they secrete immunomodulatory and neurotrophic factors; on the other hand, they directly differentiate into neurons and glial cells, significantly improving motor function recovery (Zou et al., 2020; Huang et al., 2021).

PSCs have become a major focus of research due to their ability to differentiate into cells from all three germ layers. They can be induced to produce functional cells from the endoderm, mesoderm, and ectoderm (Tian et al., 2023). As a classic pluripotent cell, embryonic stem cells can differentiate into functional, tissue-specific cells under a specific induction system (Evans and Kaufman, 1981). Transplantation studies in SCI models have confirmed that differentiated neurons and glial cells can accurately integrate into the injured area, effectively promoting the reconstruction of neural circuits (Shao et al., 2019). Germline stem cells also show neural differentiation potential. For example, spermatogonial stem cells can trans-differentiate into functional neurons under specific culture conditions (Glaser et al., 2008). These findings not only reveal the diversity of stem cell mechanisms (including cell replacement, paracrine regulation, and exosome-mediated molecular delivery) but also lay a theoretical foundation for the development of multi-target combined treatment strategies.

The application of stem cell therapy in SCI has developed a multi-level research system from basic studies to clinical translation. Human PSCs have shown significant potential for neural repair in preclinical studies due to their strong neural differentiation ability (Farzaneh et al., 2020). In the non-human primate (common marmoset) SCI model, transplantation of human induced PSC-derived neural precursor cells not only achieves non-tumorigenic differentiation but also significantly improves motor function through axon regeneration mediated by new neurons (Nagoshi et al., 2020). However, clinical translation still faces significant challenges. Existing clinical trial data show that stem cell transplantation therapies (e.g., MSCs, adipose-derived MSCs) have good safety profiles but have not demonstrated significant functional improvements (Oh et al., 2016; Bydon et al., 2024). This discrepancy between preclinical research and clinical application may be due to factors such as mismatches in stem cell survival rate, transplantation timing, and microenvironment regulation, as well as the lack of effective treatment strategies (Yamazaki et al., 2020).

Optimizing the stem cell delivery system (e.g., biomaterial scaffold loading, targeted homing technology) or implementing combined treatment strategies (e.g., co-delivery with neurotrophic factors, electrical stimulation synergy) may help overcome the current treatment bottleneck. These findings provide an important theoretical basis for developing a precise and time-controlled stem cell treatment system.

## 3 Ferroptosis and SCI

Ferroptosis is an iron-dependent form of programmed cell death driven by phospholipid peroxidation. The core mechanism involves the inactivation of *GPX4* or inhibition of the cystine/glutamate antiporter (*System Xc^−^*), leading to the collapse of antioxidant defenses, accumulation of lipid peroxidation-derived reactive oxygen species (ROS), and ultimately, oxidative cell death (Li et al., 2020; Liu et al., 2022; Tang et al., 2021). This process relies on the peroxidation of polyunsaturated fatty acids and free radical chain reactions involving iron ions. Ferroptosis plays physiological roles in tumor suppression, immune surveillance, and the elimination of drug-resistant cancer cells. It is also involved in pathological processes, including inflammation, neurodegenerative diseases, and ischemia–reperfusion injury. Its regulatory network encompasses redox homeostasis, iron metabolism, lipid metabolism, and mitochondrial activity (Jiang et al., 2021; Sun et al., 2020; Wu et al., 2021; Chen et al., 2021).

**Figure 2:**
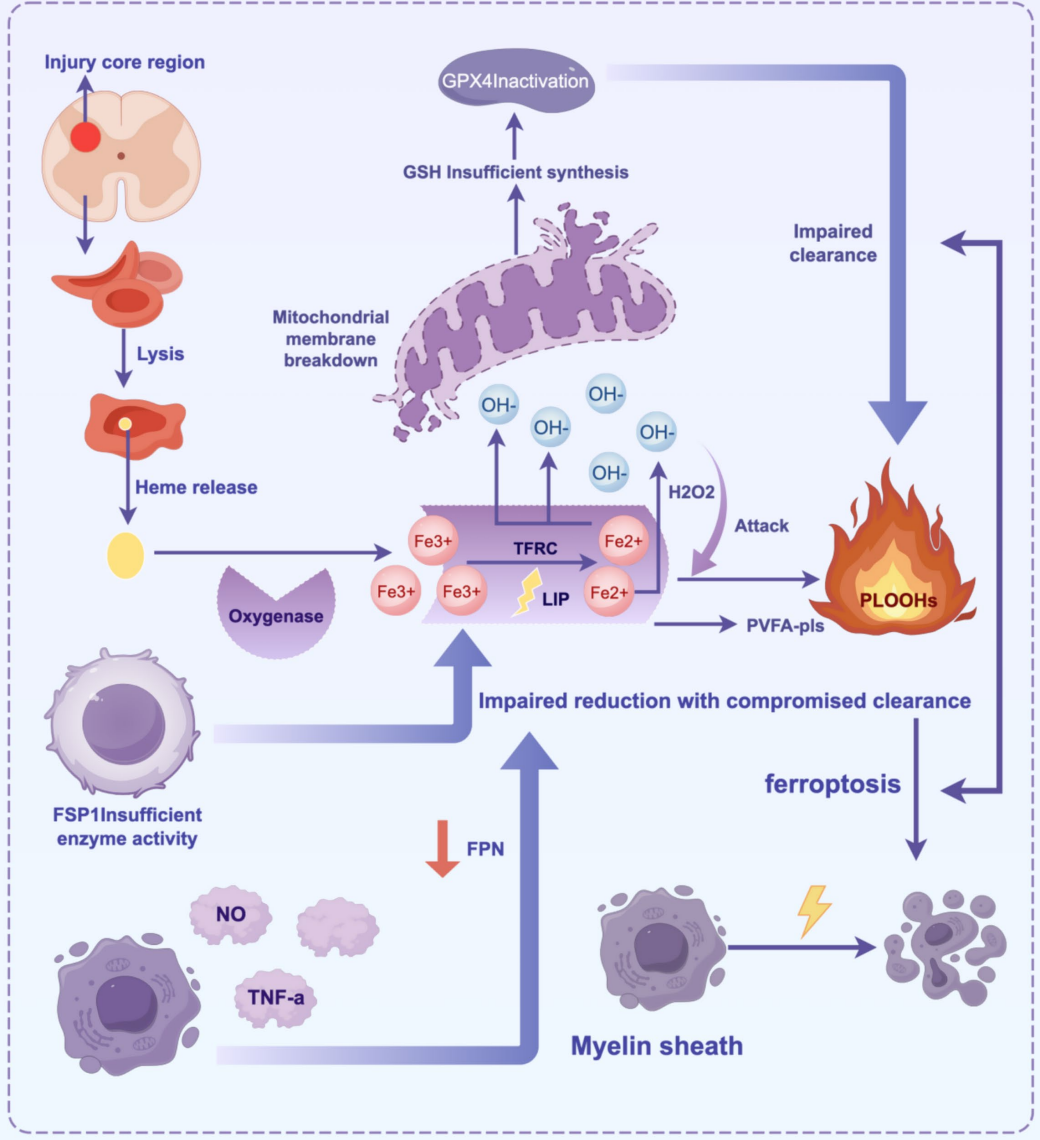
The core mechanism of ferroptosis in SCI involves the cascade of “iron overload-lipid peroxidation-antioxidant imbalance-inflammatory cycle”.

In the pathological process of SCI, ferroptosis exacerbates secondary neural injury through a multi-step mechanism. Local bleeding from the primary injury leads to red blood cell rupture and the release of heme, which is degraded to generate free iron (Fe^3+^). This iron enters the cell through transferrin receptor (*TFRC*) endocytosis and is reduced to Fe^2+^ (Ohgami et al., 2005), forming an unstable labile iron pool (Breuer et al., 2008). Fe^2+^ catalyzes the generation of hydroxyl radicals (·OH) through the Fenton reaction, triggering lipid peroxidation of polyunsaturated fatty acid phospholipids (PUFA-PLs). This generates lipid peroxides (PLOOHs), which disrupt cell membrane integrity and release damage-associated molecular patterns (DAMPs), further activating inflammatory responses (Reis and Spickett, 2012; Valko et al., 2005). Simultaneously, dysfunction of *System Xc^−^* leads to insufficient glutathione (GSH) synthesis, resulting in the loss of GSH-dependent *GPX4* activity and an inability to reduce PLOOHs (Zhang et al., 2021; Zheng and Conrad, 2020). The insufficient compensation of the Ferroptosis inhibitory protein 1-ubiquinone pathway further exacerbates the lipid free radical chain reaction. In the inflammatory microenvironment, microglia release nitric oxide (NO) and proinflammatory factors (e.g., *TNF-α*), upregulate iron uptake-related proteins, and inhibit iron export, establishing a vicious cycle of “iron overload-oxidative stress-inflammation” (Feng et al., 2021). Oligodendrocytes, which are rich in PUFA-PLs, are highly sensitive to ferroptosis. Their death leads to myelin disintegration and white matter damage, hindering nerve conduction. Experimental studies have shown that iron chelators (e.g., deferoxamine) inhibit the Fenton reaction by reducing free iron levels. Additionally, zinc enhances antioxidant defenses by activating the *NRF2* pathway, and targeting Acyl-CoA synthetase long-chain family member 4 (*ACSL4*) or supplementing monounsaturated fatty acids can regulate lipid metabolism. These strategies significantly improve neurological function recovery in animal models and provide potential targets for clinical intervention (Li et al., 2022; Figure 2).

Primary injury leads to bleeding, and red blood cells release heme, which degrades into Fe^3+^. This iron is endocytosed and reduced to Fe^2+^ by *TFRC*, forming an unstable LIP. Fe^2+^ generates OḤ through the Fenton reaction, which attacks membrane PUFA-PLs, triggering lipid peroxidation and generating PLOOHs. This destroys membrane structure and releases DAMPs, which activate inflammation. The collapse of the antioxidant system occurs when *System Xc^−^* dysfunction reduces GSH synthesis, and *GPX4* inactivation prevents the clearance of PLOOHs. The *FSP1-CoQ10* pathway provides insufficient compensation, aggravating oxidative damage. Activated microglia in the inflammatory microenvironment release NO/TNF-α, upregulate *TFRC*, and inhibit *FPN*, forming a cycle of “iron accumulation → oxidative stress → inflammation.” Oligodendrocytes are sensitive to ferroptosis due to the high concentration of PUFA-PLs in myelin. Their death triggers demyelination and white matter damage (Table 1).

**Table 1:** Research progress on ferroptosis-related molecular targets in SCI.

Intervention method	Target	*in vivo* efficacy	References
Knockout *USP11*	Autophagy regulation by Beclin 1	*In vivo* knockout of *USP11* inhibited the autophagy activation of Beclin 1 and significantly reduced neuronal cell ferroptosis.	Rong et al. (2022)
Celastrol	*NRF2/xCT/GPX4* pathway	Celastrol can repair SCI by inhibiting ferroptosis through upregulating the *NRF2/xCT/GPX4* axis.	Shen et al. (2024)
Zinc	*NRF2/GPX4* pathway	Zinc promotes the degradation of oxidative stress products and lipid peroxides through the *NRF2/HO-1* and *GPX4* signaling pathways, thereby inhibiting neuronal ferroptosis.	Ge et al. (2021)
ADSC-Exos	*NRF2/SLC7A11/GPX4* pathway	ADSC-Exos may inhibit ferroptosis after SCI through the *NRF2/SLC7A11/GPX4* pathway and promote the recovery of vascular and neural functions.	Wu et al. (2024)
Dihydroorotate dehydrogenase	*P53/ALOX15* pathway	Dihydroorotate dehydrogenase can inhibit the *P53/ALOX15* signaling pathway, thereby inhibiting lipid peroxidation and neuronal ferroptosis, ultimately alleviating SCI.	Li et al. (2023)
SYVN1	*HMGB1/NRF2/HO-1* axis	SYVN1 overexpression can alleviate spinal cord ischemia–reperfusion injury by downregulating HMGB1 and promoting *NRF2*/HO-1 pathway activation	Guo et al. (2023)
EPO	*xCT/GPX4* pathway	EPO mediates ferroptosis inhibition by upregulating xCT and GPX4, thereby improving limb function recovery and spinal cord regeneration after SCI.	Kang et al. (2023)
Metformin	*NRF2* Pathway	Met treatment can reduce malondialdehyde content, regulate inflammatory factor levels, activate nuclear factor E2-related factor 2 signaling pathway, and improve long-term efficacy by improving motor dysfunction caused by SCI.	Wang et al. (2022)
TMP	*GPX4/ACSL4* axis	TMP alleviates neuronal ferroptosis by regulating the *GPX4*/*ACSL4* axis, thereby protecting the remaining neurons around the injury site, reducing glial scar formation, and promoting functional recovery.	Liu et al. (2024)

USP11, ubiquitin-specific protease 11; ADSC-Exos, adipose-derived stem cell exosomes; GPX4, glutathione peroxidase 4; xCT, cystine/glutamate antiporter; SYVN1, synoviolin 1; ASCL4, Acyl-CoA synthetase long-chain family member 4; ALOX15, arachidonate 15-lipoxygenase; EPO, erythropoietin; HMGB1, high mobility group box 1; TMP, Tetramethylpyrazine.

## 4 Application of stem cell therapy targeting ferroptosis in SCI

The previous article described the mechanism of ferroptosis after spinal cord injury. Concurrently, studies have shown that ferroptosis is an important pathological process of neuronal death after spinal cord injury. Once iron overload, ROS accumulation, lipid peroxidation or characteristic mitochondrial changes occur in cells, ferroptosis will occur rapidly (Berndt et al., 2024; Zheng et al., 2024). Therefore, inhibiting lipid peroxidation and iron overload can be an important way to treat spinal cord injury by targeted control of ferroptosis. Stem cells can interfere with ferroptosis through complex molecular networks, including RNA regulation, mitochondrial repair, and activation of key signaling pathways, inhibiting lipid peroxidation and iron overload, thereby inhibiting ferroptosis and promoting spinal cord injury repair. The following article will describe the specific mechanism of stem cell targeted control of ferroptosis to treat spinal cord injury.

Recent studies have demonstrated that stem cell-derived exosomes play a multi-target role in inhibiting ferroptosis after SCI by delivering non-coding RNA or regulating key signaling pathways (Li et al., 2023). For example, long non-coding RNA lncGm36569, enriched in mesenchymal stem cell exosomes (MSC-exos), binds to miR-5627-5p, relieving its transcriptional inhibition of *FSP1* and enhancing the antioxidant capacity of neurons (Shao et al., 2022). In microRNA-mediated mechanisms, miR-219-5p delivered by bone marrow mesenchymal stem cell exosomes (BMSC-exos) inhibits the ubiquitination degradation of *NRF2* by targeting the ubiquitin-binding enzyme UBE2Z. This stabilizes *NRF2* protein expression and activates the downstream *SLC7A11/GPX4* axis, reducing lipid peroxidation (Dong et al., 2024). Another study showed that miR-26a-5p delivered by BMSC-exos inhibited the expression of Enhancer of Zeste Homolog 2, promoting the activation of the BDNF/TrkB signaling pathway and the phosphorylation of cAMP response element-binding protein. Additionally, miR-26a-5p upregulated the K^+^-Cl^−^ co-transporter 2, which significantly improved Lipopolysaccharide-induced Pheochromocytoma Cell Line 12 cell injury and promoted spinal cord function recovery (Chen et al., 2024). At the circular RNA level, Shao et al. generated hypoxia-induced adipose-derived stem cell-derived exosomes (ADSC-exo) by pre-treating ADSCs under hypoxic conditions (93% N₂, 5% CO₂, 2% O₂). These exosomes specifically carried circ-Wdfy3, which acted as a molecular sponge, adsorbing miR-423-3p and preventing its degradation of *GPX4* mRNA. This intervention significantly reduced ROS accumulation and inflammatory factors (Shao et al., 2024). In terms of signaling pathway regulation, Wen et al. demonstrated that BMSC-exos inhibited the IL-17 pathway, reduced levels of Fe^2+^, malondialdehyde, and ROS in the SCI model, upregulated the expression of GSH, *GPX4*, and cystine/glutamate antiporter, and downregulated the long-chain family member 4 of acyl-CoA synthetase. This combination of actions synergistically inhibited ferroptosis and promoted functional recovery (Tang et al., 2024).

This non-coding RNA and pathway-based regulatory network is highly specific in terms of spatiotemporal control, enabling intervention at key points in the ferroptosis process. It provides a promising strategy for the precise treatment of SCI.

Mitochondria, crucial for regulating energy metabolism in eukaryotic cells, also play an indispensable role in cell proliferation, differentiation, immune responses, and redox balance (Nunnari and Suomalainen, 2012; Ni et al., 2015). In response to various physiological signals or external stimuli, mitochondria have developed a complex set of mitochondrial quality control (MQC) mechanisms, encompassing mitochondrial biogenesis, dynamics, and autophagy (Liu et al., 2024; Ashrafi and Schwarz, 2013). MQC is essential for cells to cope with internal and external stresses, maintaining mitochondrial function and homeostasis. This process involves multiple regulatory levels, including mitochondrial-nuclear communication (mitochondrial retrograde signaling), changes in mitochondrial morphology (mitochondrial dynamics), and selective removal of damaged mitochondria (mitochondrial autophagy) (Al Amir Dache and Thierry, 2023). Through these mechanisms, cells can sense and respond to stress, reshaping the mitochondrial network and removing dysfunctional mitochondria, thereby ensuring systemic mitochondrial function and intracellular homeostasis (Roca-Portoles and Tait, 2021; Rahman and Quadrilatero, 2021).

MSCs transfer functional mitochondria to injured neurons through intercellular tunneling nanotubes (TNTs), directly intervening in MQC imbalance. This process is crucial for maintaining mitochondrial homeostasis and promoting neuronal survival (Hsu et al., 2016). After SCI, neurons experience ferroptosis due to excessive mitochondrial fission and abnormal mitochondrial autophagy. Single-cell transcriptome analysis revealed significant upregulation of neuronal ferroptosis markers, such as *TFRC* and 4-Hydroxynonenal, while mitochondrial morphology showed shrinkage and cristae destruction. MSC-derived mitochondria restored mitochondrial dynamic balance by fusing with the neuronal mitochondrial network, inhibiting excessive autophagy mediated by PINK1/Parkin. *In vitro* experiments demonstrated that MSC co-culture significantly reduced mitochondrial ROS in neurons, restored membrane potential and ATP levels, and decreased lipid peroxidation products (MDA) and free iron content. *In vivo*, the transplantation of MSCs improved neuronal mitochondrial morphology and promoted motor function recovery through TNT-dependent mitochondrial transfer. The TNT inhibitor cytochalasin D completely reversed this effect. This mechanism reveals a novel way for MSCs to inhibit neuronal ferroptosis at the sub-organelle level by repairing mitochondrial function and reducing the expression of ferroptosis-related markers, offering an experimental basis for neuroprotective strategies targeting mitochondrial metabolism (Yao et al., 2023).

ADSC-Exos activated *NRF2* nuclear translocation, upregulated *System Xc^−^* expression, promoted GSH synthesis, and enhanced *GPX4* activity to clear lipid peroxides. In the oxygen–glucose deprivation/reperfusion-induced endothelial cell injury model, ADSC-Exos treatment reduced intracellular ROS, increased GSH levels, and reversed the abnormal accumulation of MDA, a marker of ferroptosis. Immunofluorescence and Western blot analysis revealed that ADSC-Exos significantly upregulated *NRF2* nuclear expression and the downstream SLC7A11/*GPX4* pathway. *NRF2* inhibition with ML385 blocked these effects, confirming the critical role of this pathway in inhibiting ferroptosis. *In vivo*, ADSC-Exos promoted angiogenesis in the SCI area and improved motor function. This mechanism synergistically inhibited ferroptosis at the molecular level by enhancing cystine uptake, GSH metabolism, and lipid peroxidation repair, providing a new strategy for targeted vascular-neuron repair (Wu et al., 2024). BMSC-exos reduce *ACSL4* expression and ROS levels by inhibiting the IL-17 signaling pathway. Studies have shown that IL-17 pathway activation after SCI promotes *ACSL4*-mediated lipid peroxidation, leading to neuronal ferroptosis. Following BMSC-exo intervention, the expression of IL-17 downstream inflammatory factors (such as IL-17A, IL-17RA, Act1) decreased, as did *ACSL4* protein levels and ROS generation. *In vitro* experiments further demonstrated that IL-17 neutralizing antibodies could mimic the effects of BMSC-exos (Tang et al., 2024).

## 5 Biomaterials synergy

While many studies have confirmed that stem cells can intervene in ferroptosis and promote SCI neuron repair, their effectiveness is often limited by the local microenvironment. Therefore, understanding the stem cell microenvironment and developing a biomaterial synergy with stem cell engineering delivery systems are critical for improving the success rate of SCI repair treatments (Pennings et al., 2018; Papa et al., 2020; Kubinová, 2020).

The shear-thinning terephthalic acid (TPA)@Laponite hydrogel, loaded with dental pulp stem cells (DPSCs), removes ROS and regulates synaptic balance through both physical and chemical effects (Ying et al., 2023). The hydrogel demonstrates excellent physical properties, allowing it to remain in the body for an extended period. Its shear-thinning properties enable it to adapt to the mechanical microenvironment of the spinal cord, remove lipid ROS in the injury area, inhibit lipid peroxidation, and regulate the balance between inhibitory and excitatory neurons. This is achieved through the secretion of neurotrophic factors by DPSCs, promoting SCI recovery. While TPA@Laponite hydrogel can reduce the entanglement of blood vessels and fibrous scars, it cannot significantly promote vascular function recovery. In contrast, DPSCs can reduce fibrous scar formation, differentiate into neural and vascular cells, and aid in vascular function restoration. *In vivo* experiments revealed that combined treatment reduced muscle spasms caused by excessive excitation and promoted motor function recovery.

Small extracellular vesicles-loaded N-acryloylglycinamide/gelatin methacrylate/laponite/tannic acid hydrogel (sEVs-NGL/T) integrates the antioxidant and anti-inflammatory properties of tannic acid with the mechanical support function of laponite to construct a three-dimensional scaffold that offers sustained release and bioactivity. The hydrogel is characterized by a porous network structure, good degradation stability, and a low initial degradation rate, providing long-lasting mechanical support. Its moderate swelling properties prevent compression of surrounding tissues after implantation. *In vitro* experiments demonstrated that sEVs-NGL/T hydrogel exhibited significant antioxidant capacity in a simulated peroxidative microenvironment, effectively scavenging DPPH free radicals and reducing H₂O₂ concentration. Additionally, it enhanced the ROS protection of PC12 cells through the continuous release of sEVs derived from mesenchymal stem cells. In a rat model of spinal cord complete transection, the implantation of sEVs-NGL/T significantly improved motor function recovery, reduced cystic cavity formation at the injury site, and promoted neural tissue repair by inhibiting excessive astrocyte proliferation and encouraging nerve fiber regeneration. Furthermore, sEVs-NGL/T synergistically alleviated the inflammatory response by regulating the ROS microenvironment, reducing the levels of lipid peroxidation products (4-Hydroxynonenal and 8-Hydroxy-2′-deoxyguanosine), and inhibiting the release of proinflammatory cytokines (TNF-*α*, IL-6, IL-1β). The sustained release of sEVs enhanced the local anti-inflammatory and antioxidant effects (Liu et al., 2022). Studies have shown that human umbilical mesenchymal stem cells (Huc-MSCs) can improve the inflammatory microenvironment of SCI and promote nerve regeneration. However, due to their lack of inherent targeting ability and rapid clearance by immune cells, their efficacy is limited by the local SCI microenvironment (Wu et al., 2019; Bonilla et al., 2021). Therefore, the researchers developed a synergistic Huc-MSCs and ferroptosis inhibitor nanoparticle sustained-release system. The ROS-sensitive nanosystem, mPEG-b-Lys-BECI-TCO, anchors Huc-MSCs through a CD44 targeting sequence (Tz-A6 peptide) and loads a novel ferroptosis inhibitor, Feb-1. In the SCI microenvironment, high ROS concentrations trigger the degradation of nanocarriers and the release of Feb-1, in combination with Huc-MSC transplantation therapy. Experimental results showed that this combined therapy significantly promoted the expression of *GPX4* and xCT signaling pathways, reduced neuronal loss, and improved motor function recovery in rats by inhibiting ferroptosis and inflammatory responses. Furthermore, Western blot analysis revealed that the expression of proinflammatory factors (iNOS and IL-1β) was significantly reduced in the combined treatment group, and immunofluorescence staining confirmed increased survival of neurons in the injury area (Hua et al., 2024).

## 6 Clinical trial cases

### 6.1 Clinical trial cases and efficacy differences

A phase I study for subacute spinal cord injury (Akhlaghpasand et al., 2024) showed that 9 patients received intrathecal injection of human umbilical cord mesenchymal stem cells (HUC-MSCs) exosomes and no treatment-related adverse events occurred. The efficacy evaluation showed that the American Spinal Injury Association (ASIA) scale score improved 6 months (37.89 ± 20.65, *p* = 0.066) and 12 months (38.22 ± 20.95, *p* = 0.066) after injection compared with the baseline (36.22 ± 20.92), but due to the small sample size, the efficacy conclusion still needs to be interpreted with caution. Among patients with chronic spinal cord injury, 3 patients received combined transplantation treatment (Zamani et al., 2022). No motor function recovery was observed in the two-year follow-up, which only confirmed the safety of the treatment. The efficacy of a phase III trial (Oh et al., 2016) showed that only 2 of the 16 patients followed up had improved upper limb motor grade, and the remaining 14 showed no improvement in the 6-month follow-up, accounting for only 12.5% of the patients. This shows that autologous MSCs transplantation has limited improvement in functional recovery after spinal cord injury, further highlighting the efficacy bottleneck of single therapy. Current clinical trials generally have problems such as small sample size and inconsistent efficacy evaluation tools, resulting in the effectiveness of stem cell therapy has not been fully verified. In the future, larger-scale controlled clinical trials are needed to verify the effectiveness (Jiang et al., 2013).

### 6.2 Key barriers to clinical translatio

Most of the current research conclusions are quite different from those of animal experiments. The reason is that some studies believe that the period, site, and dose of stem cell transplantation have a great impact on prognosis. For example, acute application will expose stem cells to cytotoxic environments such as excitatory transmitters, reactive oxygen species, and inflammatory molecules, which will affect the survival rate of stem cells. Some injured parts can be injected with stem cells, but they are not suitable for stem cell survival due to low blood perfusion, while the normal proximal spinal cord above the injured part is at risk of re-injury due to high tissue pressure (Oh et al., 2016). However, this is only a conjecture and speculation stage, which needs to be further verified by control experiments, and is also one of the directions for future exploration of stem cell transplantation therapy. In addition, allogeneic stem cells may trigger immune rejection reactions, and the long-term tumorigenic risk (such as teratoma) still needs more follow-up data support.

## 7 Outlook and conclusion

The stem cell-targeted ferroptosis strategy has opened a new avenue for SCI treatment. By intervening in the key pathways of ferroptosis through multiple targets, this approach demonstrates significant neuroprotective and regenerative potential. When combined with a biomaterial delivery system, it can further enhance stem cell survival and targeting efficiency, overcoming the limitations of the local microenvironment and enabling precise, time- controlled treatments. In addition, exosome-mediated RNA regulation and mitochondrial transfer mechanisms offer innovative strategies for developing cell-free therapies, which may help circumvent the ethical and safety concerns associated with traditional stem cell transplantation.

Although preclinical studies have confirmed the therapeutic potential of stem cell-targeted ferroptosis strategies in animal models, clinical translation still faces substantial challenges, including a lack of clinical trial data and insufficient evidence of efficacy (Oh et al., 2016; Akhlaghpasand et al., 2024; Zamani et al., 2022; Shin et al., 2015). Due to the physiological and anatomical differences between animal models and human SCI, the immune responses also vary. These differences encompass factors such as SCI level, severity, timing, stem cell heterogeneity, low survival rates post-transplantation, inhibitory effects from the local microenvironment, and long-term tumorigenic risks. These issues have yet to be systematically addressed, creating a significant gap between basic research and clinical application (Lukovic et al., 2014; Lukovic et al., 2012).

Future research should focus on the following areas: (1) deepening the study of ferroptosis regulation and stem cell mechanisms; (2) clarifying the spatiotemporal expression patterns and synergistic effects of key molecules; (3) optimizing stem cell engineering strategies to enhance their antioxidant, anti-inflammatory, and exosome secretion capabilities through gene editing; (4) improving survival rates; (5) promoting multimodal treatments, such as stem cell-material-electrical stimulation synergy, while integrating microenvironment regulation and functional reconstruction (Jiang et al., 2013; Li et al., 2024); (6) For ethical optimization of alternative cell sources, it is recommended to give priority to adult stem cells with less ethical controversy to reduce dependence on embryonic stem cells (Lo and Parham, 2009).

Clinical translation faces several challenges: (1) stem cell heterogeneity leads to fluctuations in efficacy, and a standardized quality control system must be established; (2) differences between animal models and human pathology could undermine treatment efficacy, requiring verification through organoids or non-human primate models; and (3) long-term safety concerns (e.g., tumorigenicity and immune rejection) and large-scale preparation processes need further improvement (Shin et al., 2015). (4) The standardization of the ethical review framework and the ethical transparency of clinical transformation require the establishment of an interdisciplinary ethical review mechanism covering the legitimacy of stem cell sources and safety assessment of gene editing technology (Barker et al., 2018); emphasis is placed on improving the transparency and social acceptance of stem cell therapy and reducing ethical resistance in technology promotion through the disclosure of clinical trial data, optimization of the patient informed consent process, and public scientific education (King and Perrin, 2014; McCaughey et al., 2016). Despite these obstacles, with the continued integration of precision medicine and regenerative technologies, the stem cell-targeted ferroptosis strategy holds great promise for bridging the gap from laboratory research to clinical application, offering breakthrough treatment options for SCI patients in the future.
